# Predictors of severe sepsis-related in-hospital mortality based on a multicenter cohort study: The Focused Outcomes Research in Emergency Care in Acute Respiratory Distress Syndrome, Sepsis, and Trauma study

**DOI:** 10.1097/MD.0000000000024844

**Published:** 2021-02-26

**Authors:** Akiyoshi Hagiwara, Noriko Tanaka, Yosuke Inaba, Satoshi Gando, Atsushi Shiraishi, Daizoh Saitoh, Yasuhiro Otomo, Hiroto Ikeda, Hiroshi Ogura, Shigeki Kushimoto, Joji Kotani, Yuichiro Sakamoto, Yasukazu Shiino, Shin-ichiro Shiraishi, Kiyotsugu Takuma, Takehiko Tarui, Ryosuke Tsuruta, Taka-aki Nakada, Toru Hifumi, Kazuma Yamakawa, Naoshi Takeyama, Norio Yamashita, Toshikazu Abe, Masashi Ueyama, Kohji Okamoto, Junichi Sasaki, Tomohiko Masuno, Toshihiko Mayumi, Seitaro Fujishima, Yutaka Umemura, Satoshi Fujimi

**Affiliations:** a Department of Emergency Medicine, Niizashiki Chuo General Hospital, Saitama; b Biostatistics Section, Department of Data Science, Clinical Science Center, National Center for Global Health and Medicine, Tokyo; c Division of Acute and Critical Care Medicine, Department of Anesthesiology and Critical Care Medicine, Hokkaido University Graduate School of Medicine, Sapporo; d Department of Acute and Critical Care Medicine, Sapporo Higashi Tokushima Hospital, Sapporo; e Emergency and Trauma Center, Kameda Medical Center, Chiba; f Division of Traumatology, Research Institute, National Defense Medical College, Tokorozawa; g Trauma and Acute Critical Care Center, Medical Hospital, Tokyo Medical, and Dental University; h Department of Emergency Medicine, Teikyo University School of Medicine, Tokyo; i Department of Traumatology and Acute Critical Medicine, Osaka University Graduate School of Medicine, Osaka; j Division of Emergency and Critical Care Medicine, Tohoku University Graduate School of Medicine, Miyagi; k Department of Disaster and Emergency Medicine, Kobe University Graduate School of Medicine, Kobe; l Emergency and Critical Care Medicine, Saga University Hospital, Saga; m Department of Acute Medicine, Kawasaki Medical School, Kurashiki; n Department of Emergency and Critical Care Medicine, Aizu Chuo Hospital, Aizuwakamatsu; o Emergency and Critical Care Center, Kawasaki Municipal Kawasaki Hospital, Kawasaki, Kanagawa; p Department of Trauma and Critical Care Medicine, Kyorin University School of Medicine, Mitaka; q Advanced Medical Emergency & Critical Care Center, Yamaguchi University Hospital, Ube; r Department of Emergency and Critical Care Medicine, Chiba University Graduate School of Medicine, Chiba; s Department of Emergency and Critical Care Medicine, St. Luke's International Hospital, Chūō, Tokyo; t Division of Trauma and Surgical Critical Care, Osaka General Medical Center, Osaka; u Advanced Critical Care Center, Aichi Medical University Hospital, Aichi; v Advanced Emergency Medical Service Center, Kurume University Hospital, Kurume, Fukuoka; w Department of General Medicine, Juntendo University, Tokyo; x Department of Trauma, Critical Care Medicine, and Burn Center, Japan Community Healthcare Organization, Chukyo Hospital, Nagoya, Aichi; y Department of Surgery, Center for Gastroenterology and Liver Disease, Kitakyushu City Yahata Hospital; z Department of Emergency and Critical Care Medicine, Keio University School of Medicine, Shinjuku City; aa Department of Emergency and Critical Care Medicine, Nippon Medical School, Bunkyo City, Tokyo; ab Department of Emergency Medicine, School of Medicine, University of Occupational and Environmental Health, Kitakyushu, Fukuoka; ac Center for General Medicine Education, Keio University School of Medicine, Shinjuku City, Tokyo; ad Department of Traumatology and Acute Critical Medicine, Osaka University Graduate School of Medicine; ae Division of Trauma and Surgical Critical Care, Osaka General Medical Center, Japan.

**Keywords:** Acute Physiology and Chronic Health Evaluation, in-hospital mortality, Japan, sepsis, Sequential Organ Failure Assessment, structural equation model

## Abstract

This study aimed to identify prognostic factors for severe sepsis-related in-hospital mortality using the structural equation model (SEM) analysis with statistical causality. Sepsis data from the Focused Outcomes Research in Emergency Care in Acute Respiratory Distress Syndrome, Sepsis, and Trauma study (FORECAST), a multicenter cohort study, was used. Forty seven observed variables from the database were used to construct 4 latent variables. SEM analysis was performed on these latent variables to analyze the statistical causality among these data. This study evaluated whether the variables had an effect on in-hospital mortality. Overall, 1148 patients were enrolled. The SEM analysis showed that the 72-hour physical condition was the strongest latent variable affecting mortality, followed by physical condition before treatment. Furthermore, the 72-hour physical condition and the physical condition before treatment strongly influenced the Sequential Organ Failure Assessment (SOFA) score with path coefficients of 0.954 and 0.845, respectively. The SOFA score was the strongest variable that affected mortality after the onset of severe sepsis. The score remains the most robust prognostic factor and can facilitate appropriate policy development on care.

## Abstract 2

Supplemental Digital Content is available in the text

## 1 Introduction

Risk factors of severe sepsis-related mortality have been widely reported in studies conducted in Western and other countries other than Japan.^[1–4]^ In these studies, the statistical methods to identify prognostic factors of severe sepsis employed mainly multivariate analyses. The observed variables in these analyses were often selected from the following data: patients’ basic background characteristics (such as age and sex), comorbidities (such as cancers and chronic lung disease), physical condition before treatment (such as the presence or absence of septic shock), physical conditions during treatment (such as lactic acidosis), or selected treatment procedures (such as whether fluid resuscitation, recommended in the guidelines, was performed or not); these variables were then simultaneously entered into multivariate analysis models. However, treatment and treatment responses can have complex cause-and-effect relationships or temporal relationships. While multivariate analysis can resolve the effect of confounding on the observed variables, it cannot estimate a cause-and-effect or temporal relationship. For example, when a response variable is set as in-hospital mortality, an observed variable measured at a time closer to death can predict mortality more accurately. This variable can be considered to be an intermediate variable, and the variable observed before the intermediate variable can be considered to be a confounding factor.^[5,6]^

Therefore, to estimate the effect of different variables on mortality, only data on intermediate variables can be used accurately in a model in which all the other observed variables are entered simultaneously. Although estimating the effects of data of patients’ basic background characteristics, physical conditions before treatments, or selected treatment procedures on mortality is likely to be biased, it is important to determine the extent to which these factors could affect prognosis.

The predictors of mortality that are usually evaluated are identified from previously known risk factors of pathological conditions. Because such risk factors have complex correlations, including temporal relationships, they cannot be identified using classical multivariate regression analyses.^[7]^ Therefore, to accurately determine these predictors and analyze temporal relations or causal structures, data need to be based on information obtained from large sample sets.

Structural equation modeling (SEM) is a statistical approach used to understand social and natural phenomena by introducing latent variables that cannot be directly observed and used to identify causal relationships between the latent and response variables, or between latent variables. SEM analysis was developed to analyze data that are suspected of having statistical causal structures, and SEM is widely used to test hypotheses in developmental psychology.^[8]^ Recently, studies using SEM analyses have also been reported in the field of clinical medicine.^[9–12]^ However, to date, no study has used SEM to analyze the risk factors for severe sepsis-related mortality in the field of critical care medicine.

The clinical course of patients with severe sepsis have complex cause-and-effect or temporal relationships. Therefore, we aimed to assess the clinical course of patients with severe sepsis using SEM analysis and perform an exploratory study to identify predictors of severe sepsis-related hospital mortality.

## 2 Methods

### 2.1 The Focused Outcomes Research in emergency care in acute respiratory distress Syndrome, Sepsis and Trauma (FORECAST) study

The Focused Outcomes Research in Emergency Care in Acute Respiratory Distress Syndrome, Sepsis and Trauma (FORECAST) study was a multicenter, cohort study conducted by the Japanese Association for Acute Medicine, which commenced in January 2016. Fifty nine intensive care units (ICUs) in Japan participated in the FORECAST study, which included the Acute Respiratory Distress Syndrome (ARDS), Sepsis, and Trauma study. The FORECAST sepsis study was registered with the University Hospital: Medical Information Network Clinical Trials Registry (UMIN – CTR ID: 000019742). Data were collected using the electronic data capture system of the UMIN Network. The sepsis study was completed in March 2017 when the number of cases reached the predetermined sample size (n = 1000), and data were locked on January 18, 2018.

### 2.2 Study design and setting

This study analyzed the data of patients with severe sepsis who were admitted to the 59 ICUs from April 2016 to March 2017 and who were entered into the FORECAST sepsis study database. After applying inclusion and exclusion criteria, a total of 1184 patients were enrolled in this study. The inclusion criteria were:

1. age ≥ 16 years and
2. the presence of severe sepsis according to the Sepsis-2 criteria (Supplement File 1).^[13]^

The exclusion criteria were

1. refusal to receive sustained life care,
2. history of cardiopulmonary arrest and resuscitation, and
3. being deemed unfit for participation by the principal investigator.

The observed demographic variables and background factors are shown in detail in Supplement File 2. The definitions of the derived variables are presented in detail in Supplement File 3. The primary outcome of the study was in-hospital mortality.

### 2.3 Statistical analysis

The baseline characteristics with respect to in-hospital death status were reported as frequencies and percentages or means and standard deviations. The Chi-Squared test, *t* test, and Wilcoxon rank sum test were used to compare the distributions of each characteristic between the nonsurvivor (hospital death) and survivor groups.

A multiple logistic regression analysis was performed to identify risk factors of in-hospital mortality in the nonsurvivor group. Based on the findings of previous studies and clinical experience, 27 patient characteristics observed at the first suspicion of sepsis were included in the model. The characteristics were age, sex, body mass index, activities of daily living (independent vs dependent), smoking (past, current, vs never), Charlson comorbidity index score, blood culture results (positive, pathogenic, vs contaminated), primary infectious focus (stool, ascites, sputa, pleural effusion, cerebrospinal fluid, wound, others, vs urine), presence of septic shock at the first suspicion of sepsis, time to antibiotic use from the first suspicion of sepsis (>60 minutes vs ≤60 minutes), the lactate value at the first suspicion of sepsis and the minimum lactate value within 6-hours after the first suspicion of sepsis (lactate value within 6-hours), platelet count, albumin, creatinine, glucose, C-reactive protein, prothrombin time-international normalized ratio (PT-INR), D-dimer, fibrinogen, pH, PaO_2_/FiO_2_ (PF) ratio, ARDS, Japanese Association for Acute Medicine disseminated intravascular coagulation score (JAAM DIC),^[14]^ Sequential Organ Failure Assessment (SOFA) score, and Acute Physiology and Chronic Health Evaluation (APACHE) II score.

A multiple imputed model analysis was performed when the covariables had more than 10% missing data in the case number. Multiple imputations were used to estimate missing data over 10 iterations, and the results from logistic regression analysis on each imputed dataset were averaged using Rubin rules.

### 2.4 Structural equation modeling

A structural equation modeling was created using 47 observed variables (the 27 baseline characteristics previously described and an additional 20 variables that were measured during treatment).

The FORECAST database consisted of 5 components:

1. host risk factors, data on prescribed drug use before enrollment in the study, and primary infection focus data;
2. clinical manifestations and laboratory findings at the first suspicion of sepsis before treatment;
3. clinical manifestations and laboratory findings 72 hours after the first suspicion of sepsis;
4. treatment and sepsis care bundle (recommended by the Surviving Sepsis Campaign Guidelines [SSCG] 2012); and
5. in-hospital mortality, ICU-free days, and ventilator-free days.

In this study, 4 latent variables, based on the components (1) to (4) described above, were created. Supplementary Fig. S1.0 shows the details of the structure model comprising 4 latent variables and the observed variables included in each latent variable. The latent variable included

1. “at risk of sepsis,” which indicated component (1);
2. “physical condition at the first suspicion of sepsis before treatment,” which indicated component (2);
3. “physical condition 72 hours after the first suspicion of sepsis,” which indicated component (3); and
4. “treatment and sepsis care bundle,” which indicated component (4).

“At risk of sepsis” influenced “physical condition at the first suspicion of sepsis before treatment ” and “mortality” (arrows A and B), whereas “physical condition at the first suspicion of sepsis before treatment ” influenced “ treatment and sepsis care bundle ” (arrow C), “physical condition 72 hours after the first suspicion of sepsis ” (arrow D), and “mortality” (arrow E). “Treatment and sepsis care bundle” influenced “physical condition 72 hours after the first suspicion of sepsis” (arrow F), while “physical condition 72 hours after the first suspicion of sepsis” influenced “mortality” (arrow G).

SEM was used to determine whether the hypothetical latent structural model fit the data, and which variables had strong direct or indirect effects on mortality. The full information maximum likelihood method.^[15]^ was used to estimate the parameters in the model, while the root mean square error of approximation (RMSEA) was calculated to determine the fit index for the overall model.

Supplementary Fig. S1.1, shows the original path diagram of the equation used in the analysis. A brief description of the SEM analysis is provided in Supplement File 4.

All p values were two-sided, and *P* < .05 was considered to be statistically significant. All statistical analyses were performed using R version 3.5.1 (the R Foundation for Statistical Computing, Vienna, Austria) and AMOS v. 25 (IBM SPSS, Tokyo).

## 3 Results

### 3.1 Patient characteristics

A total of 1184 patients were included in the database. Supplementary Table S1 summarizes the characteristics of these patients. After excluding 36 patients whose outcomes were not entered in the database, 1148 patients were finally included in our study. Of the 1148 patients, 269 died in the hospital, while 879 survived and were discharged. The overall mortality was 23.4% (269/1148).

### 3.2 At risk of sepsis

The factors that were significantly higher among nonsurvivors than among survivors were age (mean ± SD, 73.7 ± 11.8 vs 69.8 ± 15.1, *P* < .001), Charlson comorbidity index score (median [25th percentile, 75th percentile], 2.0 [1.0, 3.0] vs 1.0 [0.0, 2.0], *P* < .001), and the number of patients that were prescribed anticoagulant drugs and corticosteroids (14.5 vs 7.6%, *P* = .001 and 17.8 vs 10.6%, *P* = .002, respectively). The primary foci of infection were significantly different between the 2 groups (*P* < .001). However, sputum was the most commonly observed primary focus of infection in both groups (46.6% in nonsurvivors and 29.7% in survivors, respectively).

### 3.3 Physical condition at the first suspicion of sepsis before treatment

The nonsurvivor group compared with the survivor group had higher

1. numbers of patients with septic shock (74.3 vs 58.9%, *P* < .001),
2. numbers of patients who received corticosteroids (50.6 vs. 24.0%, *P* < .001),
3. lactate values at the time of the first suspicion of sepsis (5.64 ± 4.68 vs. 3.81 ± 3.04, *P* < .001), and minimum lactate values within 6-hours after the first suspicion of sepsis (4.28 ± 4.04 vs 2.51 ± 1.92, *P* < .001),
4. creatinine values (2.38 ± 2.15 vs 2.04 ± 1.97, *P* = .015),
5. PT-INR values (1.64 ± 1.14 vs 1.34 ± 0.56, *P* < .001),
6. numbers of patients diagnosed with ARDS based on the Berlin definition (26.1 vs 14.4%, *P* < .001),
7. SOFA scores (10.59 ± 3.67 vs 8.07 ± 3.69, *P* < .001),
8. SOFA cardiovascular scores (3.0 [1.0, 4.0] vs 2.0 [0.0, 4.0], *P* < .001),
9. APACHE II scores (29.07 ± 8.39 vs 21.82 ± 8.31, *P* < .001), and
10. JAAM DIC scores (4.0 [3.0, 6.0] vs 3.0 [2.0, 5.0], *P* < .001).^[10]^

In contrast, the nonsurvivor group compared with the survivor group had significantly lower

1. numbers of patients who received antibiotics within 1-hour of the first suspicion of sepsis (64.0 vs 71.7%, *P* = .018),
2. levels of albumin (2.47 ± 0.68 vs 2.72 ± 0.72, *P* < .001),
3. fibrinogen levels (405.69 ± 205.90 vs 486.20 ± 222.66, *P* < .001),
4. pH values (7.32 ± 0.14 vs 7.37 ± 0.13, *P* < .001), and
5. PF ratios (187.95, [103.10, 315.42] vs. 232.00 [136.50, 342.50], *P* = .001).

### 3.4 Physical condition 72 hours after the first suspicion of sepsis

Nonsurvivors compared with survivors had higher

1. lactate values (2.57 ± 2.96 vs 1.29 ± 0.64, *P* < .001),
2. creatinine levels (1.72 ± 1.18 vs 1.34 ± 1.40, *P* < .001),
3. glucose levels (163.84 ± 57.70 vs 144.78 ± 49.31, *P* < .001),
4. PT-INR values (1.48 ± 1.33 vs 1.17 ± 0.31, *P* < .001),
5. D-dimer levels (11.25, [4.80, 19.90] vs 7.10 [3.60, 14.40], *P* = .001),
6. C-reactive protein levels (15.22 ± 9.07 vs 13.32 ± 8.28, *P* = .005),
7. incidence of ARDS (28.7 vs 10.8%, *P* < .01),
8. SOFA scores (11.64 ± 4.41 vs 7.02 ± 3.92, *P* < .001),
9. SOFA cardiovascular scores (3.0 [0.0, 4.0] vs 0.0 [0.0, 1.5], *P* < .001), and
10. JAAM DIC scores (5.0 [4.0, 6.0] vs 4.0 [2.0, 5.0], *P* < .001).

In contrast, nonsurvivors compared with survivors had significantly lower

1. albumin levels (2.06 ± 0.52 vs 2.19 ± 0.51, *P* = .002),
2. platelet counts (8.50 ± 8.85 vs 14.38 ± 18.46, *P* < .001),
3. fibrinogen levels (377.88 ± 188.52 vs 480 ± 189.39, *P* < .001), and
4. PF ratios (248.36 ± 124.06 vs 287.75 ± 99.26, *P* < .001).

### 3.5 Logistic regression analysis of in-hospital mortality

The results of the logistic regression analysis are shown in Table 1. Age and Charlson comorbidity index score were significant risk factors (odds ratio [OR]: 1.0, 95% confidence interval [CI]: 1.01–1.04 and OR: 1.2, 95% CI: 1.05–1.28, respectively) of in-hospital mortality. In terms of the primary foci of infection, sputum, and cerebrospinal fluid were significant risk factors (OR: 1.8, 95% CI: 1.12–2.80, and OR: 3.1, 95% CI: 1.18–8.25, respectively) compared with urine. Antibiotic administration within 60 minutes of the first suspicion of sepsis was a significant risk factor (OR: 1.5, 95% CI: 1.08–2.18) compared with administration after 60 minutes. Albumin (OR: 0.7; 95% CI: 0.53–0.88), fibrinogen (OR: 1.00; 95% CI: 0.99–1.00), and PT-INR (OR: 1.4; 95% CI: 1.11–1.75) were significant risk factors of in-hospital mortality. The SOFA and APACHE II scores were also significant predictors of in-hospital mortality (OR: 1.1; 95% CI: 1.01–1.18 and OR: 1.07; 95% CI: 1.04–1.09 respectively).

**Table 1:** Patients’ characteristics at the first suspicion of sepsis: logistic regression analysis of mortality using multiple imputation.

	Odds ratio	Lower 95% CI^∗^	Upper 95% CI	*P* value
Patients’ background				
Age	1.023	1.010	1.037	.001
Sex (male vs female)	0.998	0.830	1.200	.981
Body mass index	1.029	0.997	1.062	.075
Activities of daily living, Independent vs dependent	1.027	0.843	1.251	.792
Smoking, past vs never	0.954	0.726	1.253	.734
Smoking, current vs never	0.994	0.705	1.401	.971
Charlson index	1.161	1.053	1.280	.003
Blood culture				
Blood culture results, positive vs negative	1.194	0.871	1.637	.270
Blood culture results, pathogenic vs contaminated	1.421	0.851	2.375	.180
Primary infection focus				
Stool vs urine	0.644	0.172	2.414	.515
Ascites vs urine	0.802	0.458	1.402	.439
Sputa vs urine	1.799	1.157	2.799	.010
Pleural effusion vs urine	0.772	0.193	3.078	.714
Cerebrospinal fluid vs urine	3.114	1.176	8.245	.023
Wound vs urine	1.107	0.569	2.153	.764
Others vs urine	0.795	0.481	1.314	.372
Septic shock and antibiotic administration				
Septic shock at the first suspicion of sepsis (yes vs no)	1.000	0.792	1.264	.998
Time to antibiotic use from the first suspicion of sepsis (> 60 minutes vs≤ 60 minutes)	1.532	1.078	2.176	.017
Laboratory findings				
Lactate value at the first suspicion of sepsis	0.997	0.979	1.016	.761
Minimum lactate value within 6 hours^#^	1.017	0.992	1.043	.192
Platelet count at the first suspicion of sepsis	1.009	0.995	1.022	.195
Albumin value at the first suspicion of sepsis	0.681	0.525	0.882	.004
Creatinine value at the first suspicion of sepsis	0.956	0.873	1.048	.339
Glucose value at the first suspicion of sepsis	1.001	0.999	1.002	.401
C-reactive protein value at the first suspicion of sepsis	1.009	0.989	1.029	.372
PT-INR at the first suspicion of sepsis	1.396	1.110	1.754	.004
D-dimer value at the first suspicion of sepsis	1.001	0.996	1.005	.762
Fibrinogen value at the first suspicion of sepsis	0.999	0.997	1.000	.020
Blood gas findings				
PF ratio at the first suspicion of sepsis	1.001	0.999	1.002	.273
pH value at the first suspicion of sepsis	1.280	0.318	5.158	.728
Scores				
ARDS at the first suspicion of sepsis, yes vs no	1.096	0.878	1.369	.417
JAAM DIC score at the first suspicion of sepsis	1.069	0.963	1.187	.213
SOFA score at the first suspicion of sepsis	1.092	1.012	1.179	.026
APACHE II score at the first suspicion of sepsis	1.067	1.041	1.093	<.001

APACHE = Acute Physiology and Chronic Health Evaluation, ARDS = acute respiratory distress syndrome (diagnosed by the Berlin definition), CI = confidence interval, DIC = disseminated intravascular coagulation, JAAM = Japanese Association for Acute Medicine, PF ratio = PaO2/FiO2, PT-INR = prothrombin time-international normalized ratio, SOFA = Sequential Organ Failure Assessment.

# Minimum lactate value within 6 hours; minimum value within 6 hours after the first suspicion.

∗ *P* < .05, statistical significance.

### 3.6 SEM analysis

**Figure 1:**
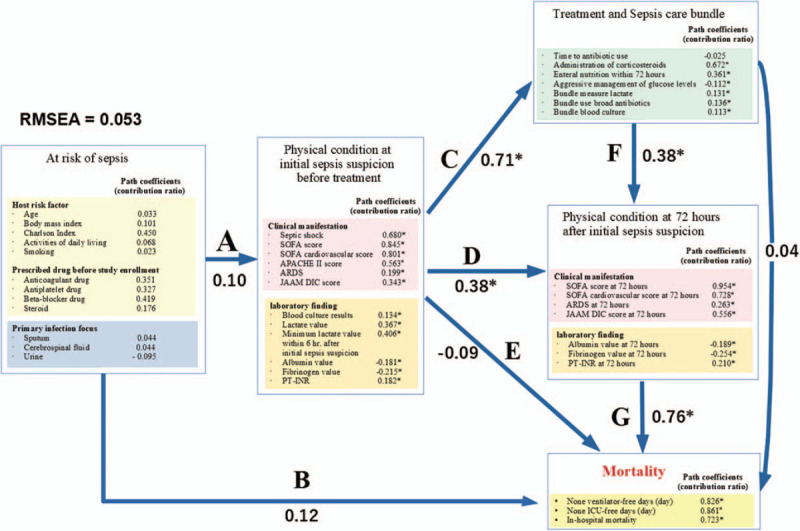
Hypothetical latent structure model and path coefficients between the latent constructs. This path diagram shows the factor structure model that represents the study's hypothesis. The titles in the rectangles show the names of the latent variables. The arrows represent the relationship between cause and effect. “At risk of sepsis” influenced “Physical condition at the first suspicion of sepsis before treatment” and “Mortality” (A and B). “Physical condition at the first suspicion of sepsis before treatment” influenced “Treatment and sepsis care bundle,” “Physical condition 72 hours after the first suspicion of sepsis,” and “Mortality” (C, D, and E). “Treatment and sepsis care bundle” influenced “Physical condition 72 hours after the first suspicion of sepsis” (F). “Physical condition 72 hours after the first suspicion of sepsis” influenced “Mortality” (G). Numerical values near Character (A, B,…, G) are path coefficients (standardized partial regression coefficient) between each latent variable. ^∗^ signifies *P* < .001. RMSEA = root mean square error of approximation.

Figure 1 and Supplementary Fig. S2 show the path coefficients (standardized partial regression coefficients) for the latent constructs in the latent structure model. The SEM was an acceptable fit for the data (RMSEA = 0.053). The SEM analysis found that “physical condition 72 hours after the first suspicion of sepsis” (direct effect: 0.76) was the strongest latent prognostic factor, followed by “physical condition at the first suspicion of sepsis before treatment” (direct effect: −0.09, indirect effect 0.52), while “at risk of sepsis” (direct: 0.12, indirect 0.04) was the weakest prognostic factor. However, the observed variables for the latent constructs “at risk of sepsis,” including age and the Charlson comorbidity index score, were found to be independently associated with in-hospital mortality based on the multiple logistic regression analysis.

Fig. 1 and Supplementary Table S2 show the path coefficients for the covariables and latent constructs that are not identical but are associated with factor scores for each latent variable. “At risk of sepsis” was influenced strongly by the Charlson comorbidity index, with a path coefficient of **γ** **=** 0.45. Furthermore, anticoagulants, antiplatelet drugs, and betablockers that were prescribed before the study enrollment had a path coefficient γ = 0.351, 0.327, and 0.419, respectively.

The “physical condition at the first suspicion of sepsis before treatment,” was strongly influenced by the SOFA score and SOFA cardiovascular score at the first suspicion of sepsis with path coefficients of γ = 0.845 and 0.801, respectively. Other factors with the path coefficient of γ ≥ 0.5 were “septic shock” and “APACHE II score,” both at the first suspicion of sepsis. The albumin and fibrinogen values showed negative path coefficients (γ = −0.181 and −0.215, respectively).

The “physical condition 72 hours after the first suspicion of sepsis” was influenced by the SOFA score at 72 hours after the first suspicion of sepsis with the highest path coefficient value of γ = 0.954. Other factors with the coefficient of ≥ 0.5 included the SOFA cardiovascular score (γ = 0.728) and the JAAM DIC score (γ = 0.556), both at 72 hours after the first suspicion of sepsis. The albumin and fibrinogen values at 72 hours after the first suspicion of sepsis showed negative path coefficients (γ = −0.189 and −0.254, respectively).

“Treatment and sepsis care bundle” were influenced by the administration of corticosteroids (path coefficient, γ = 0.672) and enteral nutrition within 72 hours (γ = 0.361). Time to antibiotic use and aggressive management of glucose levels showed negative path coefficients (γ = −0.025 and −0.112, respectively). Mortality was influenced by no ventilator-free days and no ICU-free days with a path coefficient γ = 0.826 and 0.861, respectively.

### 3.7 SEM analysis for SOFA scores and SOFA cardiovascular scores

Supplementary Figure S3 shows the path diagram excluding the observed variables other than SOFA scores and SOFA cardiovascular scores for “physical condition at the first suspicion of sepsis before treatment” and “physical condition 72 hours after the first suspicion of sepsis.” The observed variables in “at risk of sepsis” and “treatment and sepsis care bundle” remained the same as in the original path diagram. The RMSEA was 0.042, which was better than the RMSEA of the original path diagram.

## 4 Discussion

### 4.1 Latent variables in the structural equation modeling

Since the risk factors for severe sepsis have complex correlations, including temporal relationships,^[7]^ they cannot be identified using classical multivariate regression analyses. One of the interesting results from our SEM analysis with statistical causality was that “at risk of sepsis” was not associated with “physical condition at the first suspicion of sepsis before treatment” or “mortality.” These findings suggest that the host risk factor, the prescribed drug before the study enrollment, and the primary infection focus (Fig. 1) are not related to in-hospital mortality after the onset of severe sepsis. Severely septic patients with a high comorbidity index or who are of older age will have a higher mortality; however, these factors are not the cause of severe sepsis and do not have causality. The physical condition at the first suspicion of sepsis before treatment significantly influenced the treatment and sepsis care bundle as well as the physical condition 72 hours after the first suspicion of sepsis. This suggests that the course of treatment after the onset of severe sepsis can have a significant influence on patient mortality.

Therefore, risk factors for in-hospital mortality should be selected from covariables that play a role after the occurrence of sepsis. The physical condition at the first suspicion of sepsis before treatment strongly influenced the physical condition 72 hours after the first suspicion of sepsis. The physical condition 72 hours after the first suspicion of sepsis and mortality had the strongest path coefficients in the latent structure model. However, the physical condition at the first suspicion of sepsis before treatment did not influence mortality. These findings also suggest that in-hospital mortality is predominantly influenced by the patients’ physical condition during the treatment. Interestingly, while treatment and sepsis care bundle significantly influenced the physical condition 72 hours after the first suspicion of sepsis, it did not significantly influence mortality. These findings highlight the need for further development in the treatment of severe sepsis to improve the related mortality.

### 4.2 Observed variables in the structural equation modeling

The SOFA score 72 hours after initial sepsis suspicion (observed variable) was strongly influenced by the physical condition at 72 hours after initial sepsis suspicion (latent variable). The SOFA cardiovascular score at 72 hours after initial sepsis suspicion was high. The SOFA score was also strongly influenced by the physical condition at the first suspicion of sepsis before treatment. These findings suggest that the SOFA score was a very useful predictor in terms of the physical condition when sepsis was first suspected or during treatment after 72 hours.

Excluding the observed variables other than the SOFA and SOFA cardiovascular scores in the 2 latent variables “physical condition at the first suspicion of sepsis before treatment,” and “physical condition 72 hours after the first suspicion of sepsis,” the RMSEA of the path diagram was 0.42 (Supplementary Fig. S3). The goodness of fit index for this model was better than that for the original path diagram (Supplementary Fig. S2).

Therefore, we believe that the SOFA score or the SOFA cardiovascular score can sufficiently predict the in-hospital mortality after adjusting for the patient's baseline characteristics. The SEM analysis with statistical causality showed that the strongest predictor of in-hospital mortality was the SOFA score, which is consistent with the observation made by Vincent et al 20 years ago.^[16]^ Although a large number of prognostic factors and treatments have been developed for severe sepsis in the past 20 years, the SOFA score still remains an excellent predictor for in-hospital mortality. SOFA score modifications, such as the quick SOFA and delta SOFA,^[17]^ can further help in the prediction of mortality.

The path coefficient for the APACHE II scores in the latent variable “physical condition at the first suspicion of sepsis before treatment” was 0.563, which was lower than the path coefficient for the SOFA score (0.845). The APACHE score was determined within 24-hours of admission into an ICU, and therefore, during treatment. Moreover, the APACHE II score included the patients’ basic background characteristics including age and chronic health points. These might have reduced the effect of the APACHE II score on mortality (indirect effect 0.16) when compared with that of the SOFA score which had an indirect effect of 0.25. Though the APACHE II score is used as the entry criteria in many studies, the SOFA score may be the better choice, if mortality is the endpoint of the study.^[18]^

### 4.3 Structural equation modeling vs logistic regression analysis

The SEM analysis resolved some results obtained from the logistic regression analysis that differed from clinical experience. The logistic regression analysis was performed using data obtained before the initiation of treatment for severe sepsis and the results indicated that age, the Charlson comorbidity index, primary infection foci (sputa and cerebrospinal fluid), time of antibiotic administration ≤ 1-hour, PT-INR values, SOFA scores, and APACHE II scores to be significant independent risk factors for in-hospital mortality. These results are consistent with those of other studies, except for the primary infection foci and time of antibiotic administration.^[17,19–22]^ The effectiveness of the early administration of antibiotics has been reported in many papers.^[20,23–26]^ However, the results of the univariate and multivariate analyses in this study were unusual because they demonstrated a higher mortality in patients who received antibiotics within 1-hour. This can be explained by

1. the possibility that antibiotics may have been administered early due to the patient's condition being critical, which can account for the high mortality, or
2. unmeasured biases.

However, the results of the SEM analysis showed that the time of commencement of antibiotic treatment was not related to the prognosis of patients with severe sepsis; which was also previously reported by Sterling et al.^[26]^

For the “treatment and sepsis care bundle,” the path coefficient for the administration of corticosteroids was the highest, at 0.69. This suggested that the administration of corticosteroids played an important, albeit a negative role in the treatment of sepsis. This may be due to some biases. For example, many clinicians may administer corticosteroids to save the lives of patients with very severe sepsis, who are more likely to die than others. Another bias arises from the fact that since this study had the highest frequency of patients with respiratory (sputum) infections, some of these patients with influenza infections may have been treated with steroids.^[27]^ Moreover, about 9% of the patients had been previously treated with steroids, which could have increased the risk of poorer outcomes due to immunosuppression.^[28]^

### 4.4 Study limitations

This study has some limitations. First, data on the mortality items for 36 patients (3.0%) were missing. Almost all of these patients had missing data on the covariates pertaining to the 72-hours after sepsis was first suspected. If all of them were to have died within 72 hours, the path coefficient between “physical condition at the first suspicion of sepsis before treatment” and “mortality” may have changed. Second, this study was limited to tertiary hospitals in Japan, which makes the generalization of the findings difficult. Finally, due to the relatively small number of cases, SEM analysis could not be performed using all observed variables for which univariate analysis was performed.

## 5 Conclusion

Using SEM analysis with causal structures, we assessed the clinical course of patients with severe sepsis. The SOFA score remains an excellent predictor for in-hospital mortality and should be used for making clinical judgments for severe sepsis until a better score is confirmed.

## Acknowledgments

We thank all members of the Focused Outcomes Research in Emergency Care in Acute Respiratory Distress Syndrome, Sepsis and Trauma (FORECAST) and the study group of the Japanese Association for Acute Medicine.

## Author contributions

**Conceptualization:** Akiyoshi Hagiwara, Noriko Tanaka.

**Data curation:** Akiyoshi Hagiwara, Noriko Tanaka, Yosuke Inaba, Satoshi Gando, Atsushi Shiraishi, Daizoh Saitoh, Yasuhiro Otomo, Hiroto Ikeda, Hiroshi Ogura, Shigeki Kushimoto, Joji Kotani, Yuichiro Sakamoto, Yasukazu Shiino, Shin-ichiro Shiraishi, Kiyotsugu Takuma, Takehiko Tarui, Ryosuke Tsuruta, Taka-aki Nakada, Toru Hifumi, Kazuma Yamakawa, Naoshi Takeyama, Norio Yamashita, Toshikazu Abe, Masashi Ueyama, Kohji Okamoto, Junichi Sasaki, Tomohiko Masuno, Toshihiko Mayumi, Seitaro Fujishima, Yutaka Umemura, Satoshi Fujimi.

**Formal analysis:** Akiyoshi Hagiwara, Noriko Tanaka, Yosuke Inaba.

**Investigation:** Akiyoshi Hagiwara, Noriko Tanaka.

**Methodology:** Akiyoshi Hagiwara, Noriko Tanaka, Satoshi Gando, Atsushi Shiraishi, Shigeki Kushimoto, Takehiko Tarui, Toru Hifumi, Toshikazu Abe.

**Project administration:** Satoshi Gando.

**Software:** Akiyoshi Hagiwara, Yosuke Inaba.

**Supervision:** Noriko Tanaka, Yosuke Inaba, Satoshi Gando, Atsushi Shiraishi, Daizoh Saitoh, Yasuhiro Otomo, Hiroto Ikeda, Hiroshi Ogura, Shigeki Kushimoto, Joji Kotani, Yuichiro Sakamoto, Yasukazu Shiino, Shin-ichiro Shiraishi, Kiyotsugu Takuma, Takehiko Tarui, Ryosuke Tsuruta, Taka-aki Nakada, Kazuma Yamakawa, Naoshi Takeyama, Norio Yamashita, Toshikazu Abe, Masashi Ueyama, Kohji Okamoto, Junichi Sasaki, Tomohiko Masuno, Toshihiko Mayumi, Seitaro Fujishima, Yutaka Umemura, Satoshi Fujimi.

**Writing – original draft:** Akiyoshi Hagiwara.

**Writing – review & editing:** Akiyoshi Hagiwara.

## Supplementary Material

Supplemental Digital Content

## Supplementary Material

Supplemental Digital Content

## Supplementary Material

Supplemental Digital Content

## Supplementary Material

Supplemental Digital Content

## Supplementary Material

Supplemental Digital Content

## Supplementary Material

Supplemental Digital Content

## Supplementary Material

Supplemental Digital Content

## Supplementary Material

Supplemental Digital Content

## Supplementary Material

Supplemental Digital Content

## Supplementary Material

Supplemental Digital Content
