# Supplementary material for: Predictors of severe sepsis-related in-hospital mortality based on a multicenter cohort study: The Focused Outcomes Research in Emergency Care in Acute Respiratory Distress Syndrome, Sepsis, and Trauma study
Source: Medicine (Baltimore). 2021 Feb 26;100(8):e24844. doi: 10.1097/MD.0000000000024844 (PMC7909210; doi:10.1097/MD.0000000000024844)
Supplement: Supplemental Digital Content [file medi-100-e24844-s001.docx]

**Supplement File 1**

**Sepsis-2 criteria** ^1^

1) Systolic blood pressure < 90 mmHg, mean arterial pressure < 65 mmHg, or decreased blood pressure > 40 mmHg

2) Serum creatinine level > 2.0 mg/dL or diuresis < 0.5 mL/kg/h

3) Total bilirubin count > 2.0 mg/dL

4) Platelet count < 100,000 cells/mm3

5) Arterial lactate level > 2 mmol/L

6) Prothrombin time-international normalized ratio > 1.5

7) Arterial hypoxemia (PaO2/FIO2) <200 with pneumonia or PaO2/FIO2 < 250 without pneumonia

**Reference**

1. Levy MM, Fink MP, Marshall JC, et al. International Sepsis Definitions Conference. 2001 SCCM/ESICM/ACCP/ATS/SIS International Sepsis Definitions Conference. Intensive Care Med 2003;29:530–538. https://doi.org/10.1007/s00134-003-1662-x.
