# Supplementary material for: Predictors of severe sepsis-related in-hospital mortality based on a multicenter cohort study: The Focused Outcomes Research in Emergency Care in Acute Respiratory Distress Syndrome, Sepsis, and Trauma study
Source: Medicine (Baltimore). 2021 Feb 26;100(8):e24844. doi: 10.1097/MD.0000000000024844 (PMC7909210; doi:10.1097/MD.0000000000024844)
Supplement: Supplemental Digital Content [file medi-100-e24844-s002.docx]

**Supplement File 2**

1. Demographic variables and background factors

Sex, age, race, height, weight, body mass index, intensive care unit entry route, new infectious diseases suspected from patients’ medical history, presence or absence of septic shock at the date of diagnosis, diseases that caused hospitalization, comorbidities, Charlson comorbidity index ^1^, presence or absence of livelihood protection, presence or absence of non-medical insurance, activities of daily living, smoking history, medicine prescribed, and whether or not antibiotics were used within 24-h before sepsis diagnosis (if yes, was the name of the antimicrobial drug recorded?).

2. Established sepsis care protocols (bundle)

2.1 Three-hour resuscitation bundle.

Bundle (B) 1. Serum lactate obtained, B2. Broad-spectrum antibiotic given administered, B3. Blood culture samples obtained before the administration of broad-spectrum antibiotics, B4. 30 mg/kg crystalloid fluid bolus delivered (yes/cases with indication).

2.2 Six-hour resuscitation bundle.

Six-hour resuscitation bundle; vasopressors use plus re-measured lactate

B5. Vasopressor use followed by initial fluid bolus delivery if needed to maintain a mean arterial pressure ≥65 mm Hg (yes/cases with indication), B6. Central venous pressure (CVP) measured (yes/cases with indication), B7. CVP of 8 mmHg achieved (yes/cases with indication), B8. ScvO2 measured (yes/cases with indication), B9. ScvO2 70% (or SvO2 65%) achieved (yes/cases with indication), and B10. Lactate levels re-measured if initial lactate levels were elevated (yes/cases with indication).

3. Information on infections

Whether or not blood culture results were obtained, whether or not pathogenic bacteria were detected in the blood culture (if yes, were the bacteria types and names recorded?), major foci of infection, and whether or not pathogenic bacteria were detected in the main foci (if yes, were the bacteria types and names recorded? ).

4. Vital signs

Immediately before tracheal intubation

Glasgow Coma Scale evaluation, respiratory rate, pulse rate, systolic blood pressure, diastolic blood pressure, core body temperature, and urinary volume.

5. Results of blood tests

White blood cell count, immature leukocyte (%), hematocrit, hemoglobin, platelet (PLT) count, and decreasing PLT rate.

6. Results of serum chemistry

Total protein, albumin, creatinine, bilirubin, blood glucose, Na, K, lactic acid, C-reactive protein, and procalcitonin levels.

5. Results of blood coagulation test

Prothrombin time (PT), PT ratio, PT-international normalized ratio, fibrin/fibrinogen degradation products, D dimer, fibrinogen, and antithrombin III (AT III).

6. Arterial blood gas analysis

FIO2, pH, PaCO2, PaO2, base excess.

7. Airway management

Whether or not tracheal intubation was performed, whether or not mechanical ventilation was performed (if the answer is yes, record the positive end expiratory pressure), PaO2/FiO2 ratio, and whether or not acute respiratory distress syndrome was present.

8. Severity score

Systemic inflammatory response syndrome score, the Japanese Association for Acute Medicine disseminated intravascular coagulation (DIC) criteria, the International Society on Thrombosis and Hemostasis overt DIC criteria, and Sequential Organ Failure Assessment and Acute Physiology and Chronic Health Evaluation scores.

**Reference**

1. Charlson M, Szatrowski TP, Peterson J, Gold J. Validation of a combined comorbidity index. *J Clin Epidemiol* 1994;47:1245–51. https://doi.org/10.1016/0895-4356(94)90129-5
