# Supplementary material for: Predictors of severe sepsis-related in-hospital mortality based on a multicenter cohort study: The Focused Outcomes Research in Emergency Care in Acute Respiratory Distress Syndrome, Sepsis, and Trauma study
Source: Medicine (Baltimore). 2021 Feb 26;100(8):e24844. doi: 10.1097/MD.0000000000024844 (PMC7909210; doi:10.1097/MD.0000000000024844)
Supplement: Supplemental Digital Content [file medi-100-e24844-s003.docx]

**Supplement File 3**

**Definition of the derived variables**

- The date at which severe sepsis was diagnosed was derived from the date and time (hours, minutes) at which a physician suspected severe sepsis based on the database.
- The survival time was defined as the number of days of hospital stay.
- The primary infection focus was diagnosed using the culture results of the specimens obtained within 24 hours after severe sepsis was first suspected.
- In cases of suspected severe sepsis by a physician, and if the names of broad-spectrum antibiotics were entered into the database, we judged that the physician had used the antibiotics for the treatment of severe sepsis.
- The time from the physician’s first suspicion of sepsis to antibiotic administration was calculated and converted to binary data (≤ 60 minutes, > 60 minutes).
- If pathogenic bacteria were detected on blood culture testing, we judged that the patients had a bloodstream infection.
- Ventilator-free days (VFD) was defined as the number of days within the first 28 days after enrolment during which a patient was able to breathe without a ventilator. VFD in patients who died during the study period was assigned as 0.
- Intensive care unit (ICU)-free days were calculated in the same manner.
- When we used VFD- and ICU-free days in structural equation model (SEM) analysis, we transformed VFD- and “ICU-free days” into “none ventilator-free days (28 [days] –VFD)” and “none ICU-free days (28 [days] – ICU free days)”, respectively.
