# Supplementary material for: Predictors of severe sepsis-related in-hospital mortality based on a multicenter cohort study: The Focused Outcomes Research in Emergency Care in Acute Respiratory Distress Syndrome, Sepsis, and Trauma study
Source: Medicine (Baltimore). 2021 Feb 26;100(8):e24844. doi: 10.1097/MD.0000000000024844 (PMC7909210; doi:10.1097/MD.0000000000024844)
Supplement: Supplemental Digital Content [file medi-100-e24844-s004.docx]

Supplement File 4

fig. 1


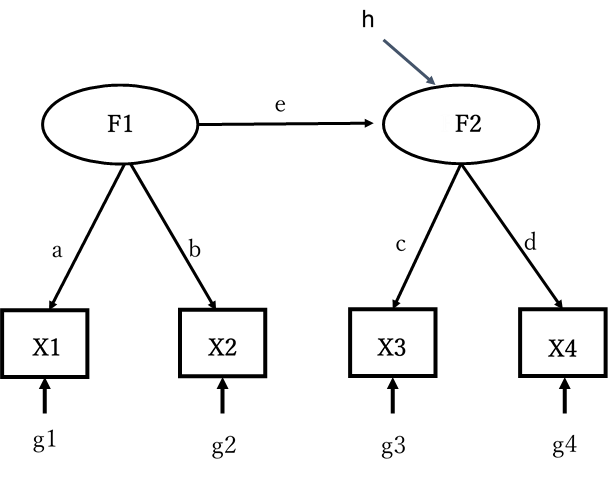


A simple model to explain SEM analysis (fig 1). Assume that are four observed variables, x1 to x4, and two latent variables F1 and F2. In this model, there is a causal path between the two latent variables (labeled e).

This model assumes that F1 influences X1 with path coefficient (a) and X2 with path coefficient (b). These relationships present a factor analysis of the F1. Similarly, F2 influences X3 with (c) and X4 with (d). These relationships present a factor analysis of the F2. Further, F1 influences F2 with path coefficient (e). The (g1) to (g4) and (h ) represent the error values of each variable

A simple equation of the relationship between F1 and F2 is shown as follows.

F2 = e*F1 + h ---------- (1)

The relationship between F1 and x1, x2 are shown as follows.

x1 = a*F1 + g1 --------- (2)

X2 = b*F1 + g2 -------- (3)

Similarly,

x3 = c*F2 --------------- (4)

x4 = d*F2 --------------- (5)

These five equations are expressed in terms of structural equations as follows.


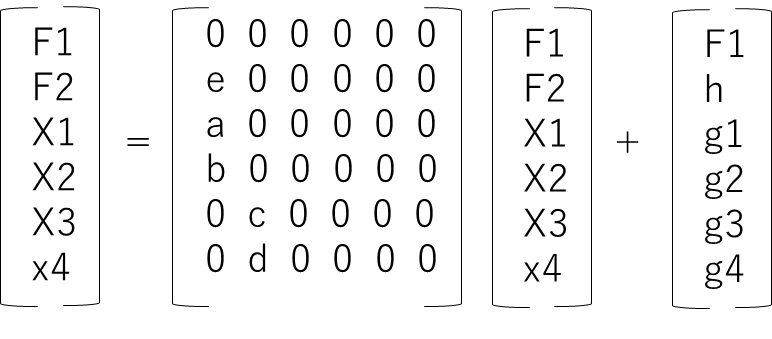


Each path coefficient’s values are calculated by maximum likelihood estimation.

When the path coefficient (e) is a large value (close to 1 in a standardization estimate), it can be said that F1 affects F2 with statistical causality. In contrast, when the path coefficient (e) is a small value (close to 0 in a standard estimate), it can be said that F1 does not affect F2. that is to say, it can be said that F1 and F2 have not statistical causality.
