# Supplementary material for: Predictors of severe sepsis-related in-hospital mortality based on a multicenter cohort study: The Focused Outcomes Research in Emergency Care in Acute Respiratory Distress Syndrome, Sepsis, and Trauma study
Source: Medicine (Baltimore). 2021 Feb 26;100(8):e24844. doi: 10.1097/MD.0000000000024844 (PMC7909210; doi:10.1097/MD.0000000000024844)
Supplement: Supplemental Digital Content [file medi-100-e24844-s005.docx]

**Supplementary Table S1** Patients’ characteristics

|  |  | **In-hospital mortality^a^** | |  |
| --- | --- | --- | --- | --- |
|  |  | **Non-survival** | **Survival** | **p value** |
| **n (number)** |  | 269 | 879 |  |
| **At risk of sepsis** |  |  |  |  |
| ***Patients’ background*** |  |  |  |  |
| Age, year, mean (SD) |  | 73.70 (11.80) | 69.83 (15.10) | <0.001 |
| Sex, n (%) | Female | 107 (39.8) | 348 (39.6) | 1.000 |
|  | Male | 162 (60.2) | 531 (60.4) |  |
| BMI, mean (SD), n =1158 |  | 22.34 (5.45) | 22.15 (5.20) | 0.61 |
| Charlson index, median  [25^th^, 75^th^ percentile] |  | 2.00 [1.00, 3.00] | 1.00 [0.00, 2.00] | <0.001 |
| ADL, n (%) | Dependent | 69 (25.7) | 205 (23.3) | 0.415 |
| n = 1182 | Independent | 199 (74.3) | 673 (76.7) |  |
| Smoking, n (%) | Current | 31 (12.9) | 114 (14.4) | 0.806 |
| n = 1068 | Former | 78 (32.4) | 243 (30.7) |  |
|  | Never | 132 (54.8) | 434 (54.9) |  |
| Anticoagulant drug prescribed before study enrollment^b^ (%) | No | 230 (85.5) | 812 (92.4) | 0.001 |
|  | Yes | 39 (14.5) | 67 (7.6) |  |
| Antiplatelet drug prescribed before study enrollment^a^ (%) | No | 238 (88.5) | 744 (84.6) | 0.137 |
|  | Yes | 31 (11.5) | 135 (15.4) |  |
| Beta-blocker drug prescribed before study enrollment^a^ (%) | No | 246 (91.4) | 807 (91.8) | 0.899 |
|  | Yes | 23 (8.6) | 72 (8.2) |  |
| Corticossteroid prescribed before study enrollment^a^ (%) | No | 221 (82.2) | 786 (89.4) | 0.002 |
|  | Yes | 48 (17.8) | 93 (10.6) |  |
| ***Primary infection focus*** |  |  |  |  |
| Primary infection focus, n (%) | Stool | 3 (1.3) | 14 (1.7) | <0.001^&^ |
| n = 1078 | Ascites | 24 (10.3) | 105 (13.0) |  |
|  | Sputum | 109 (46.6) | 240 (29.7) |  |
|  | Pleural effusion | 4 (1.7) | 9 (1.1) |  |
|  | Cerebrospinal fluid | 10 (4.3) | 12 (1.5) |  |
|  | Wound | 17 (7.3) | 57 (7.1) |  |
|  | Urine | 30 (12.8) | 200 (24.8) |  |
|  | Others | 37 (15.8) | 171 (21.2) |  |
| **Physical condition at the first suspicion of sepsis before treatment** | | | | |
| ***Blood culture and septic shock*** |  |  |  |  |
| Blood culture results, n (%) | Negative | 101 (37.7) | 366 (41.8) | 0.256 |
| n = 1179 | Positive | 167 (62.3) | 509 (58.2) |  |
| Blood culture results, pathogenic or contaminated, n (%) | Contaminated | 12 (8.1) | 55 (11.8) | 0.229 |
| n = 1125 | Pathogenic | 137 (91.9) | 413 (88.2) |  |
| Septic shock^d^ at first suspicion of sepsis (%) | No | 69 (25.7) | 361 (41.1) | <0.001 |
|  | Yes | 200 (74.3) | 518 (58.9) |  |
| ***Bundle*** |  |  |  |  |
| Bundle measure lactate^e^, n (%) | No | 5 (1.9) | 31 (3.5) | 0.23 |
| n = 1183 | Yes | 264 (98.1) | 847 (96.5) |  |
| Bundle use of broad antibiotics^e^, n (%) | No | 41 (15.3) | 150 (17.1) | 0.514 |
| n = 1182 | Yes | 227 (84.7) | 728 (82.9) |  |
| Bundle blood culture^e^, n (%) | No | 26 (9.7) | 68 (7.8) | 0.312 |
| n = 1181 | Yes | 243 (90.3) | 808 (92.2) |  |
| ***Treatment*** |  |  |  |  |
| Time to antibiotic use^f^ (min.) | > 60 min. | 96 (36.0) | 247 (28.3) | 0.018 |
| n = 1176 | <= 60 min. | 171 (64.0) | 626 (71.7) |  |
| Administration of corticosteroids, n (%) | No | 132 (49.4) | 664 (76.0) | <0.001 |
| n = 1177 | Yes | 135 (50.6) | 210 (24.0) |  |
| Enteral nutrition within 72 hours, n (%) | No | 157 (58.8) | 460 (52.7) | 0.092 |
| n = 1176 | Yes | 110 (41.2) | 413 (47.3) |  |
| Aggressive management for glucose^g^, n (%) | No | 191 (71.5) | 673 (77.2) | 0.061 |
| n = 1175 | Yes | 76 (28.5) | 199 (22.8) |  |
| ***Laboratory findings*** |  |  |  |  |
| Lactate value at first suspicion of sepsis (mmol/L), n = 1102 |  | 5.64 (4.68) | 3.81 (3.04) | <0.001 |
| Minimum lactate value within 6 hours after the suspicion (mmol/L), n = 986 |  | 4.28 (4.04) | 2.51 (1.92) | <0.001 |
| Albumin value at first suspicion of sepsis (g/dL), n = 1159 |  | 2.47 (0.68) | 2.72 (0.72) | <0.001 |
| Platelet count at first suspicion of sepsis (/uL), n =1181 |  | 16.63 (20.06) | 17.45 (12.19) | 0.418 |
| Creatinine value at first suspicion of sepsis (mg/dL), n = 1180 |  | 2.38 (2.15) | 2.04 (1.97) | 0.015 |
| Glucose value at first suspicion of sepsis (g/dL), n = 1163 |  | 164.23 (138.09) | 162.47 (100.54) | 0.82 |
| PT-INR^a^ at first suspicion of sepsis, n = 1151 |  | 1.64 (1.14) | 1.34 (0.56) | <0.001 |
| D dimer value at first suspicion of sepsis (ug/mL), n = 889 |  | 23.51 (53.31) | 18.39 (49.91) | 0.217 |
| Fibrinogen value at first suspicion of sepsis (mg/dL), n = 963 |  | 405.69 (205.90) | 486.20 (222.66) | <0.001 |
| C-reactive protein at first suspicion of sepsis (mg/dL), n = 1170 |  | 15.40 [7.50, 22.60] | 16.00 [7.70, 25.30] | 0.464 |
| pH value at first suspicion of sepsis, n = 1113 |  | 7.32 (0.14) | 7.37 (0.13) | <0.001 |
| PF ratio at first suspicion of sepsis, n = 1097 |  | 187.95 [103.10, 315.42] | 232.00 [136.50, 342.50] | 0.001 |
| ARDS at first suspicion of sepsis^$^ (%) | No | 182 (74.0) | 682 (85.6) | <0.001 |
| n = 1079 | Yes | 64 (26.0) | 115 (14.4) |  |
| ***Severity score*** |  |  |  |  |
| SOFA score at first suspicion of sepsis, n = 1008 |  | 10.59 (3.67) | 8.07 (3.69) | <0.001 |
| SOFA cardiovascular score at first suspicion of sepsis, n = 1176 |  | 3.00 [1.00, 4.00] | 2.00 [0.00, 4.00] | <0.001 |
| APACHE II score at first suspicion of sepsis, n = 1058 |  | 29.07 (8.39) | 21.82 (8.31) | <0.001 |
| JAAM DIC score at first suspicion of sepsis, n = 1021 |  | 4.00 [3.00, 6.00] | 3.00 [2.00, 5.00] | <0.001 |
| **Physical condition 72-hours after the first suspicion of sepsis** | | | | |
| ***Laboratory finding*** |  |  |  |  |
| Lactate value at 72 hours after the first suspicion of sepsis (mmol/L), n = 861 |  | 2.57 (2.96) | 1.29 (0.64) | <0.001 |
| Albumin value at 72 hours after the first suspicion of sepsis (g/dL), n = 1023 |  | 2.06 (0.52) | 2.19 (0.51) | 0.002 |
| Platelet counts at 72 hours after the first suspicion of sepsis (/uL), n = 1087 |  | 8.50 (8.85) | 14.38 (18.46) | <0.001 |
| Creatinine value at 72 hours after the first suspicion of sepsis (mg/dL), n = 1087 |  | 1.72 (1.18) | 1.34 (1.40) | <0.001 |
| Glucose value at 72 hours after the first suspicion of sepsis (g/dL), n = 1003 |  | 163.84 (57.70) | 144.78 (49.31) | <0.001 |
| PT-INR^a^ at 72 hours after the first suspicion of sepsis, n = 960 |  | 1.48 (1.33) | 1.17 (0.31) | <0.001 |
| D dimer value at 72 hours after the first suspicion of sepsis (ug/mL), n = 667 |  | 11.25 [4.80, 19.90] | 7.10 [3.60, 14.40] | 0.001 |
| Fibrinogen value at 72 hours after the first suspicion of sepsis (mg/dL), n = 772 |  | 377.88 (188.52) | 480.22 (189.39) | <0.001 |
| C-reactive protein at 72 hours after the first suspicion of sepsis (mg/dL), n = 1043 |  | 15.22 (9.07) | 13.32 (8.28) | 0.005 |
| pH value at 72 hours after the first suspicion of sepsis, n = 859 |  | 7.39 (0.10) | 7.53 (2.60) | 0.439 |
| PF ratio at 72 hours after the first suspicion of sepsis, n = 858 |  | 248.36 (124.06) | 287.75 (99.26) | <0.001 |
| ARDS at 72 hours after the first suspicion of sepsis^h^ (%) | No | 127 (71.3) | 621 (89.2) | <0.001 |
| n = 910 | Yes | 51 (28.7) | 75 (10.8) |  |
| ***Severity score*** |  |  |  |  |
| SOFA score at 72 hours after the first suspicion of sepsis, n = 832 |  | 11.64 (4.41) | 7.02 (3.92) | <0.001 |
| SOFA cardiovascular score at 72 hours after the first suspicion of sepsis, n = 1088 |  | 3.00 [0.00, 4.00] | 0.00 [0.00, 1.50] | <0.001 |
| JAAM DIC score at 72 hours after the first suspicion of sepsis, n = 807 |  | 5.00 [4.00, 6.00] | 4.00 [2.00, 5.00] | <0.001 |
| **Prognosis** |  |  |  |  |
| Ventilator-free days (day)^i^ , n = 1172 |  | 0 | 24.00 [18.00, 28.00] |  |
| ICU-free days (day)^i^, n = 956 |  | 0 | 20.00 [12.00, 24.00] |  |

*BMI* body mass index, *ADL* activities of daily living, *SD* standard deviation, *ICU* intensive care unit, *PF* PaO2/FiO2, *ARDS* acute respiratory distress syndrome, *PT-INR* prothrombin time-international normalized ratio, *JAAM* Japanese Association for Acute Medicine, *DIC* disseminated intravascular coagulation, *SOFA* sequential organ failure assessment, *APACHE* Acute Physiology and Chronic Health Evaluation.

^a^Thirty-six observations were missing in-hospital mortality values.

^b^Did the patient receive the antibiotics prescribed before study enrollment?

^c^This number denotes the missing value in patients with positive blood culture results.

^d^Septic shock was diagnosed by SSCG 2012 criteria; and arterial hypotension was defined as systolic blood pressure < 90 mm Hg, mean arterial pressure < 70 mm Hg, or a systolic blood pressure decrease > 40 mm Hg in adults, or less than two standard deviations.

^e^The bundle indicates the treatment policy including treatment initiation within 3 or 6 hours from admission, as strongly recommended by SSCG 2012.

^f^Time to antibiotic use: Time from suspected sepsis to antibiotic administration.

^g^Was the glucose value controlled to ≤ 180 mg/dL from 6 to 24 hours after the first diagnosis?

^h^ARDS was diagnosed according to the Berlin definition.

^i^Ventilator -free days (VFD) was defined as the number of days within the first 28 days after enrolment during which a patient was able to breathe without a ventilator. VFD in patients who died during the study period was assigned as 0. Intensive care unit (ICU)-free days were calculated in the same manner.

The p value was calculated using the Pearson’s chi-squared test
