# Supplementary material for: Predictors of severe sepsis-related in-hospital mortality based on a multicenter cohort study: The Focused Outcomes Research in Emergency Care in Acute Respiratory Distress Syndrome, Sepsis, and Trauma study
Source: Medicine (Baltimore). 2021 Feb 26;100(8):e24844. doi: 10.1097/MD.0000000000024844 (PMC7909210; doi:10.1097/MD.0000000000024844)
Supplement: Supplemental Digital Content [file medi-100-e24844-s006.docx]

**Supplementary Table S2** Path coefficients between covariables and latent constructs: Results of factor analysis for each latent construct

| **Covariable (*x*)^a^** |  | **Latent construct (ξ)** | **Path coefficients (γ)** | |  |  |
| --- | --- | --- | --- | --- | --- | --- |
|  |  |  | **Standardized**  **estimation** | **Non-standardized**  **estimation** | **Standard error** | **p values** |
| Age | = | At risk of sepsis | 0.033 | 1 | NA | NA |
| Body mass index | = | // | 0.101 | 1.1 | 0.72 | 0.13 |
| Charlson Index | = | // | 0.450 | 1.54 | 0.786 | 0.05 |
| Activities of daily living | = | // | 0.068 | 0.061 | 0.053 | 0.25 |
| Smoking | = | // | 0.023 | 0.035 | 0.071 | 0.621 |
| Anticoagulant drug^a^ | = | // | 0.351 | 0.213 | 0.109 | 0.052 |
| Antiplatelet drug^a^ | = | // | 0.327 | 0.242 | 0.125 | 0.053 |
| Beta-blocker drug^a^ | = | // | 0.419 | 0.241 | 0.123 | 0.051 |
| Steroid^a^ | = | // | 0.176 | 0.120 | 0.067 | 0.072 |
| ***Primary infection focus*** |  |  |  |  |  |  |
| Sputum | = | // | 0.044 | 0.043 | 0.049 | 0.373 |
| Cerebrospinal fluid | = | // | 0.044 | 0.014 | 0.016 | 0.381 |
| Urine | = | // | -0.095 | -0.082 | 0.056 | 0.145 |
| Septic shock at the first suspicion of sepsis, yes or no | = | Physical condition at initial sepsis suspicion before treatment | 0.680 | 0.065 | 0.004 | < 0.001^*^ |
| SOFA score at the first suspicion of sepsis | = | // | 0.845 | 0.656 | 0.037 | <0.001^*#^ |
| SOFA cardiovascular score at the first suspicion of sepsis | = | // | 0.801 | 0.279 | 0.016 | < 0.001^*^ |
| APACHE II score at the first suspicion of sepsis | = | // | 0.563 | 1 | NA | NA |
| ARDS at the first suspicion of sepsis | = | // | 0.199 | 0.015 | 0.002 | < 0.001^*^ |
| JAAM DIC score at the first suspicion of sepsis | = | // | 0.343 | 0.145 | 0.015 | < 0.001^*^ |
| Blood culture results, pathogenic or contaminated | = | // | 0.134 | 0.013 | 0.003 | < 0.001^*^ |
| Lactate value at the first suspicion of sepsis (mmol/L) | = | // | 0.367 | 0.263 | 0.025 | < 0.001^*^ |
| Minimum lactate value within 6 h after the first suspicion (mmol/L) | = | // | 0.406 | 0.215 | 0.019 | < 0.001^*^ |
| Albumin value at the first suspicion of sepsis (g/dL) | = | // | -0.181 | -0.026 | 0.004 | < 0.001^*^ |
| Fibrinogen value at the first suspicion of sepsis (mg/dL) | = | // | -0.215 | -9.419 | 1.49 | < 0.001^*^ |
| PT-INR at the first suspicion of sepsis | = | // | 0.182 | 0.027 | 0.005 | < 0.001^*^ |
| SOFA score at 72 h after the first suspicion of sepsis | = | Physical condition at 72 h after initial sepsis suspicion, | 0.954 | 3.89 | 0.148 | < 0.001^*^ |
| SOFA cardiovascular score at 72 h after the first suspicion of sepsis | = | // | 0.728 | 1 | NA | NA |
| ARDS at 72 h after the first suspicion of sepsis | = | // | 0.263 | 0.08 | 0.009 | < 0.001^*^ |
| JAAM DIC score at 72 h after the first suspicion of sepsis | = | // | 0.556 | 1.03 | 0.064 | < 0.001^*^ |
| Albumin level at 72 h after the first suspicion of sepsis (g/dL) | = | // | -0.189 | -0.083 | 0.014 | < 0.001^*^ |
| Fibrinogen level at 72 h after the first suspicion of sepsis (mg/dL) | = | // | -0.254 | -42.098 | 5.882 | < 0.001^*^ |
| PT-INR at 72 h after the first suspicion of sepsis | = | // | 0.210 | 0.121 | 0.019 | < 0.001^*^ |
| Time to antibiotic use^b^ (min) | = | Treatment and bundle | -0.025 | -61.628 | 88.034 | 0.484 |
| Administration of corticosteroids | = | // | 0.672 | 1 | NA | NA |
| Enteral nutrition within 72 h | = | // | 0.361 | 0.596 | 0.118 | < 0.001^*^ |
| Aggressive management of glucose levels | = | // | -0.112 | -0.155 | 0.058 | 0.007^*^ |
| Bundle measure lactate | = | // | 0.131 | 0.074 | 0.021 | < 0.001^*^ |
| Bundle use broad antibiotics | = | // | 0.136 | 0.163 | 0.045 | < 0.001^*^ |
| Bundle blood culture | = | // | 0.113 | 0.099 | 0.033 | 0.002^*^ |
| None ventilator-free days (day)^c^ | = | Mortality | 0.826 | 31.116 | 1.372 | < 0.001^*^ |
| None ICU-free days (day)^c^ | = | // | 0.861 | 1 | NA | NA |
| In-hospital mortality | = | // | 0.723 | 29.485 | 1.476 | < 0.001^*^ |

“//” means “same as above”

Covariable (x) = Standardized estimation (γ) × Latent construct (ξ) + δ, “δ" denotes the error of x

^a^ Prescribed drug before study enrollment

^b^Time to antibiotic use: Time from the first suspicion of sepsis to antibiotic administration

^c^The number of “none ventilator-free days” and “none ICU-free days” were calculated as follows: 28 (days) – ventilator free days (days), and 28 (days) – ICU free days (days), respectively.

*p < 0.05, statistical significance
