# Supplementary figures and images for: Predictors of severe sepsis-related in-hospital mortality based on a multicenter cohort study: The Focused Outcomes Research in Emergency Care in Acute Respiratory Distress Syndrome, Sepsis, and Trauma study
Source: Medicine (Baltimore). 2021 Feb 26;100(8):e24844. doi: 10.1097/MD.0000000000024844 (PMC7909210; doi:10.1097/MD.0000000000024844)

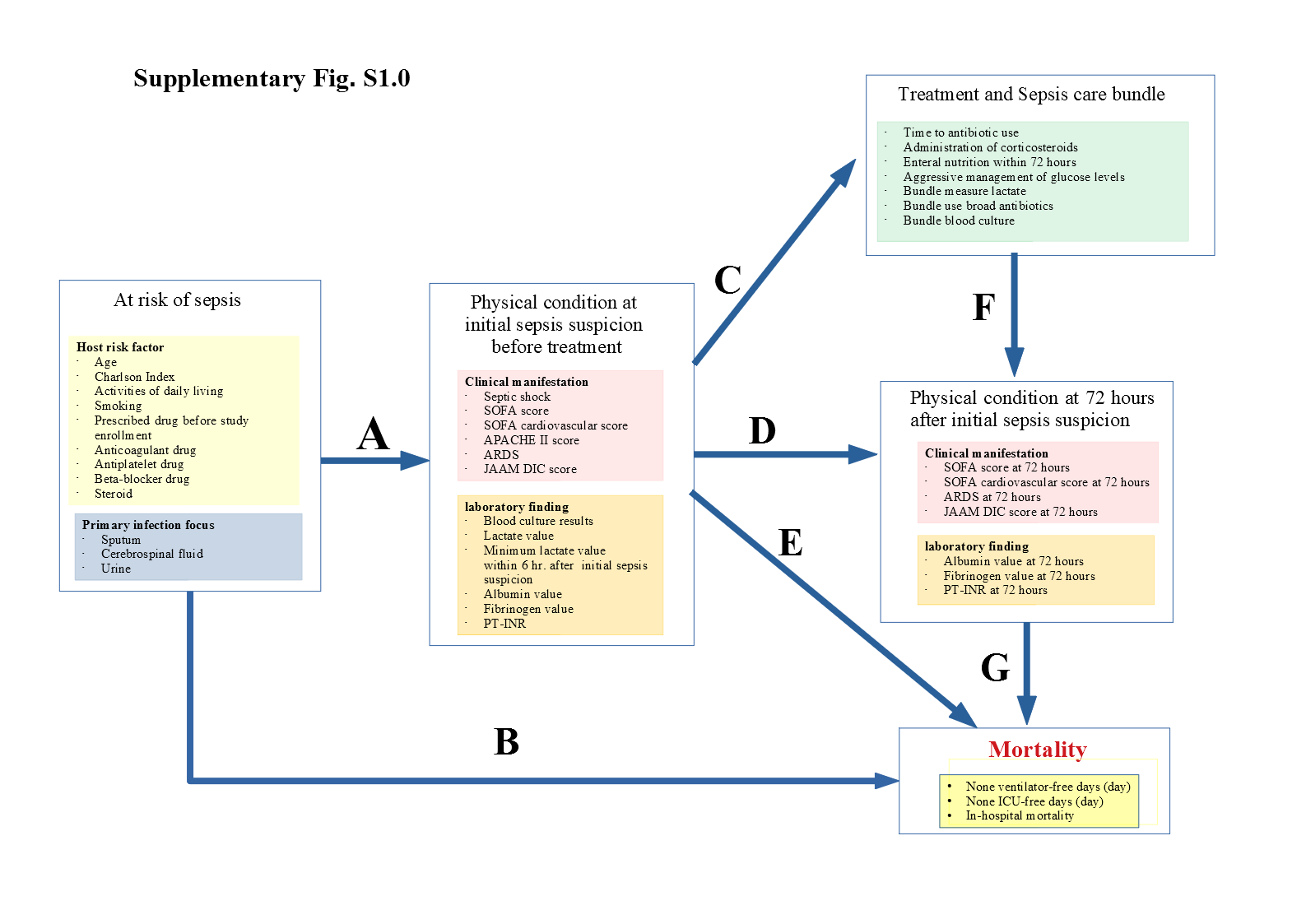

Supplement: Supplemental Digital Content [file medi-100-e24844-s007.tif]

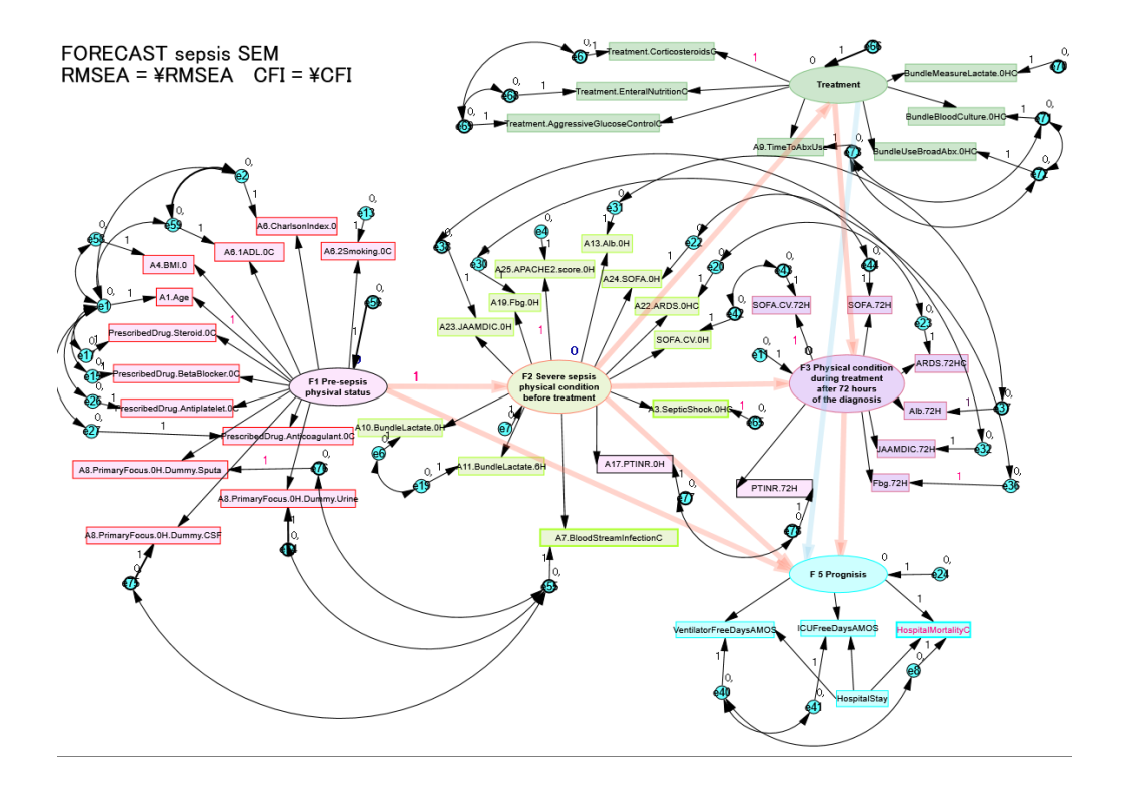

Supplement: Supplemental Digital Content [file medi-100-e24844-s008.tif]

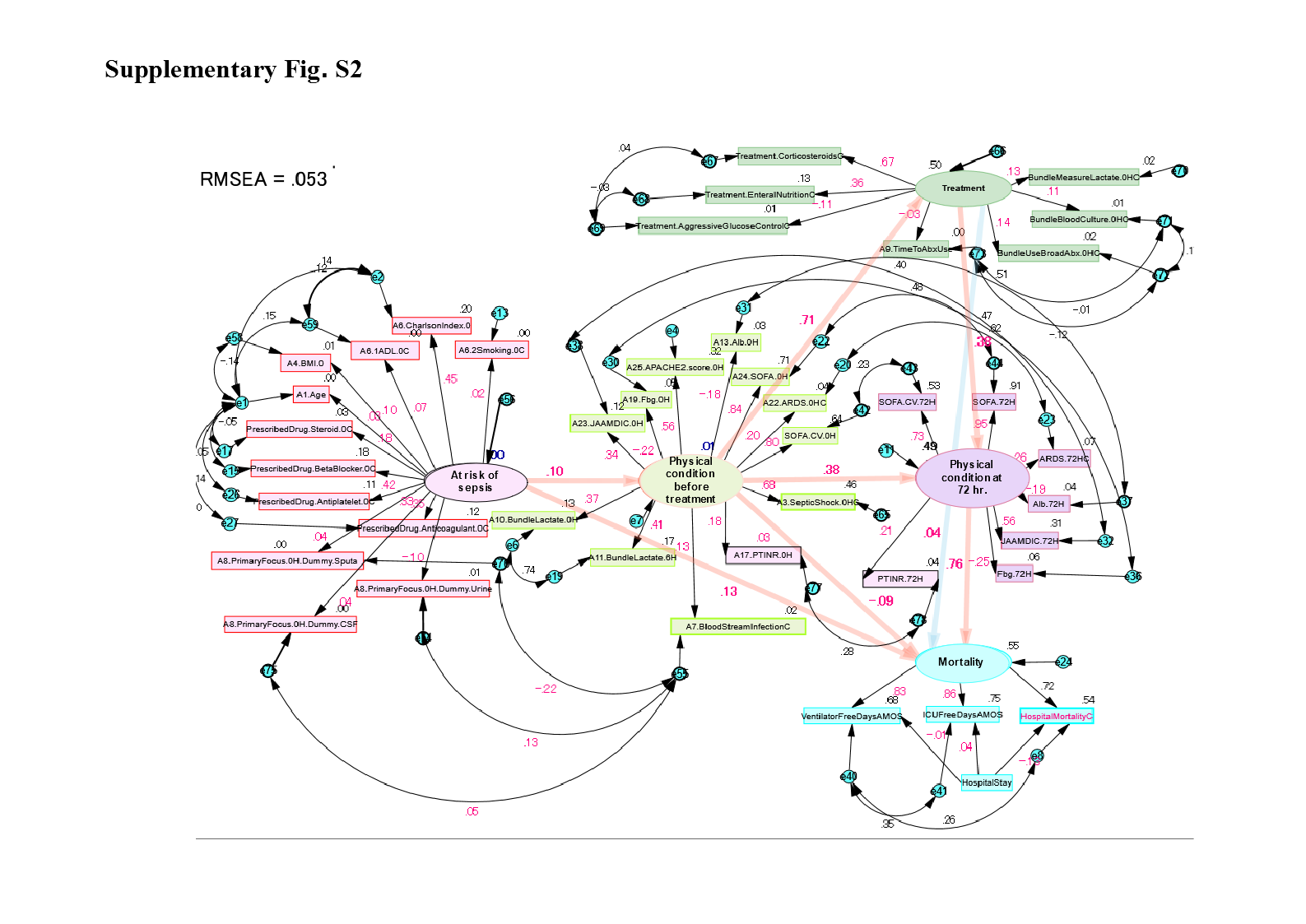

Supplement: Supplemental Digital Content [file medi-100-e24844-s009.tif]

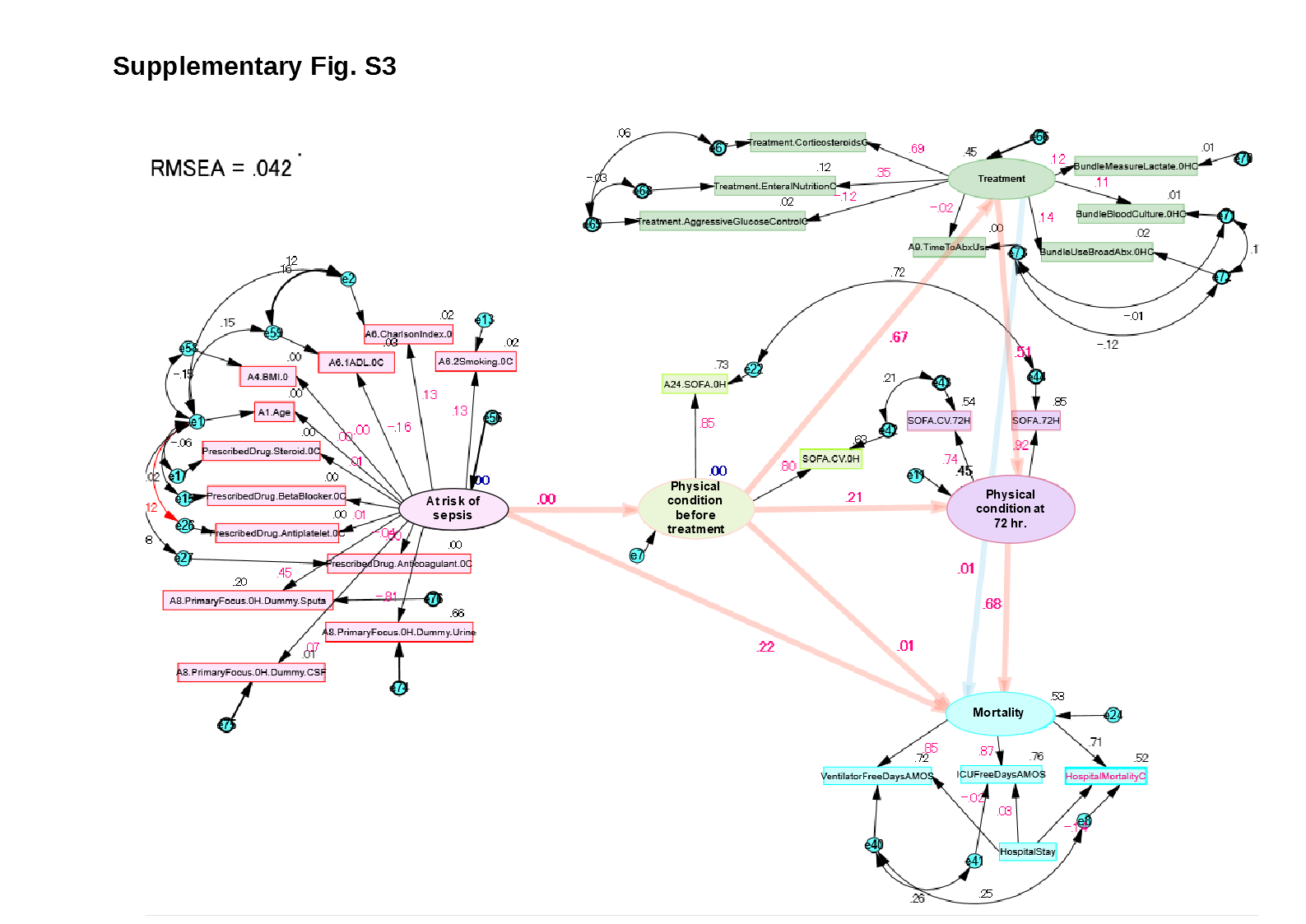

Supplement: Supplemental Digital Content [file medi-100-e24844-s010.tif]
